# Major in-hospital complications after catheter ablation of cardiac arrhythmias: individual case analysis of 43 031 procedures

**DOI:** 10.1093/europace/euad361

**Published:** 2023-12-15

**Authors:** Lars Eckardt, Florian Doldi, Omar Anwar, Nele Gessler, Katharina Scherschel, Ann-Kathrin Kahle, Aenne S von Falkenhausen, Raffael Thaler, Julian Wolfes, Andreas Metzner, Christian Meyer, Stephan Willems, Julia Köbe, Philipp Sebastian Lange, Gerrit Frommeyer, Karl-Heinz Kuck, Stefan Kääb, Gerhard Steinbeck, Moritz F Sinner

**Affiliations:** Department for Cardiology II: Electrophysiology, University Hospital Münster, Albert-Schweitzer -Campus 1, 48149 Münster, Germany; Department for Cardiology II: Electrophysiology, University Hospital Münster, Albert-Schweitzer -Campus 1, 48149 Münster, Germany; Asklepios Hospital St.Georg, Department of Cardiology and Internal Intensive Care Medicine, Faculty of Medicine, Semmelweis University Campus Hamburg, Hamburg, Germany; DZHK (German Center for Cardiovascular Research), partner site Hamburg/Kiel/Lübeck, Berlin, Germany; Asklepios Hospital St.Georg, Department of Cardiology and Internal Intensive Care Medicine, Faculty of Medicine, Semmelweis University Campus Hamburg, Hamburg, Germany; DZHK (German Center for Cardiovascular Research), partner site Hamburg/Kiel/Lübeck, Berlin, Germany; Klinik für Kardiologie, Angiologie, Intensivmedizin, cNEP Research Consortium EVK, Düsseldorf, Germany; Klinik für Kardiologie, Angiologie, Intensivmedizin, cNEP Research Consortium EVK, Düsseldorf, Germany; Department of Cardiology, University Hospital, LMU Munich, Munich, Germany; German Center for Cardiovascular Research (DZHK), partner site: Munich Heart Alliance, Munich, Germany; Department of Cardiology, University Hospital, LMU Munich, Munich, Germany; German Center for Cardiovascular Research (DZHK), partner site: Munich Heart Alliance, Munich, Germany; Department for Cardiology II: Electrophysiology, University Hospital Münster, Albert-Schweitzer -Campus 1, 48149 Münster, Germany; DZHK (German Center for Cardiovascular Research), partner site Hamburg/Kiel/Lübeck, Berlin, Germany; Klinik und Poliklinik für Kardiologie, Universitäres Herz- und Gefäßzentrum UKE Hamburg, Hamburg, Germany; Klinik für Kardiologie, Angiologie, Intensivmedizin, cNEP Research Consortium EVK, Düsseldorf, Germany; Asklepios Hospital St.Georg, Department of Cardiology and Internal Intensive Care Medicine, Faculty of Medicine, Semmelweis University Campus Hamburg, Hamburg, Germany; DZHK (German Center for Cardiovascular Research), partner site Hamburg/Kiel/Lübeck, Berlin, Germany; Department for Cardiology II: Electrophysiology, University Hospital Münster, Albert-Schweitzer -Campus 1, 48149 Münster, Germany; Department for Cardiology II: Electrophysiology, University Hospital Münster, Albert-Schweitzer -Campus 1, 48149 Münster, Germany; Department for Cardiology II: Electrophysiology, University Hospital Münster, Albert-Schweitzer -Campus 1, 48149 Münster, Germany; Asklepios Hospital St.Georg, Department of Cardiology and Internal Intensive Care Medicine, Faculty of Medicine, Semmelweis University Campus Hamburg, Hamburg, Germany; DZHK (German Center for Cardiovascular Research), partner site Hamburg/Kiel/Lübeck, Berlin, Germany; Department of Cardiology, University Hospital, LMU Munich, Munich, Germany; German Center for Cardiovascular Research (DZHK), partner site: Munich Heart Alliance, Munich, Germany; Department of Cardiology, University Hospital, LMU Munich, Munich, Germany; German Center for Cardiovascular Research (DZHK), partner site: Munich Heart Alliance, Munich, Germany; Department of Cardiology, University Hospital, LMU Munich, Munich, Germany; German Center for Cardiovascular Research (DZHK), partner site: Munich Heart Alliance, Munich, Germany

**Keywords:** Interventional electrophysiology, Catheter ablation, Complications, Atrial flutter, Atrial fibrillation, Ventricular tachycardia, Multicentric analysis

## Abstract

**Aims:**

In-hospital complications of catheter ablation for atrial fibrillation (AF), atrial flutter (AFL), and ventricular tachycardia (VT) may be overestimated by analyses of administrative data.

**Methods and results:**

We determined the incidences of in-hospital mortality, major bleeding, and stroke around AF, AFL, and VT ablations in four German tertiary centres between 2005 and 2020. All cases were coded by the G-DRG- and OPS-systems. Uniform code search terms were applied defining both the types of ablations for AF, AFL, and VT and the occurrence of major adverse events including femoral vascular complications, iatrogenic tamponade, stroke, and in-hospital death. Importantly, all complications were individually reviewed based on patient-level source records. Overall, 43 031 ablations were analysed (30 361 AF; 9364 AFL; 3306 VT). The number of ablations/year more than doubled from 2005 (*n* = 1569) to 2020 (*n* = 3317) with 3 times and 2.5 times more AF and VT ablations in 2020 (*n* = 2404 and *n* = 301, respectively) as compared to 2005 (*n* = 817 and *n* = 120, respectively), but a rather stable number of AFL ablations (*n* = 554 vs. *n* = 612). Major peri-procedural complications occurred in 594 (1.4%) patients. Complication rates were 1.1% (*n* = 325) for AF, 1.0% (*n* = 95) for AFL, and 5.3% (*n* = 175) for VT. With an increase in complex AF/VT procedures, the overall complication rate significantly increased (0.76% in 2005 vs. 1.81% in 2020; *P* = 0.004); but remained low over time. Following patient-adjudication, all in-hospital cardiac tamponades (0.7%) and strokes (0.2%) were related to ablation. Major femoral vascular complications requiring surgical intervention occurred in 0.4% of all patients. The in-hospital mortality rate adjudicated to be ablation-related was lower than the coded mortality rate: AF: 0.03% vs. 0.04%; AFL: 0.04% vs. 0.14%; VT: 0.42% vs. 1.48%.

**Conclusion:**

Major adverse events are low and comparable after catheter ablation for AFL and AF (∼1.0%), whereas they are five times higher for VT ablations. In the presence of an increase in complex ablation procedures, a moderate but significant increase in overall complications from 2005–20 was observed. Individual case analysis demonstrated a lower than coded ablation-related in-hospital mortality. This highlights the importance of individual case adjudication when analysing administrative data.

## Graphical Abstract

**Graphical Abstract:**
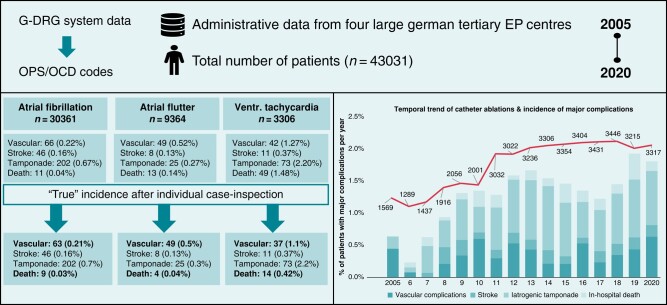

**What’s new?**

- From 2005 to 2020, an increase in complex procedures with low and slightly increasing complication rates was observed.
- Individual case analysis demonstrated a lower than coded ablation-related in-hospital mortality than reported by previous studies.
- Major adverse events are comparable after catheter ablation for typical atrial flutter and atrial fibrillation, whereas they are five times higher for ablation of ventricular tachycardias.
- When analysing ablation-associated complications, individual case inspection is critical to provide correct incidences of ablation-associated complications.

## Introduction

Catheter ablation of cardiac arrhythmias is established as a pivotal part in the treatment of supraventricular and ventricular arrhythmias. It is considered first-line therapy in the treatment of paroxysmal supraventricular tachycardias^1^ and idiopathic ventricular tachycardias.^2,3^ In addition, prognostic benefits of early catheter ablation of atrial fibrillation (AF)^4^ and ventricular tachycardia (VT) have been shown by recent studies.^5,6^ This is in line with an increasing number of catheter ablations each year.^7–9^ The more elective catheter ablations are the more important it becomes to lower complications to a minimum.^10^ It is hence of great relevance to gain further understanding of procedure-related complications.^11^

Incidences of in-hospital complications tend to vary by type of arrhythmia, the complexity of the ablation, patient comorbidities, and the experience of the performing centre. Due to higher arrhythmia complexity and a more advanced comorbidity profile including heart failure catheter ablations of VT are associated with higher peri-interventional complications than ablations of right isthmus dependent atrial flutter (AFL) or AF ablations.^12–16^ Hosseini *et al*.^15^ reported data from the Nationwide Inpatient Sample (NIS) database on patients with AF and AFL demonstrating an overall complication incidence of 7.2% and 3.9% for AF and AFL ablations, respectively. Interestingly, in German-wide administrative data analyses, Steinbeck *et al*.^17^ and König *et al*.^18^ recognized a significantly higher incidence of in-hospital mortality after cavo-tricuspid isthmus (CTI) ablation for AFL, which is usually considered a rather safe ablation,^19^ as compared to AF ablation. Therefore, we aimed at analysing major peri-interventional complications including in-hospital mortality with an individual case analysis from catheter ablation procedures for typical AFL, AF, and VT performed at four high-volume ablation centres from 2005 through 2020.

## Methods

### Data acquisition

**Figure 1:**
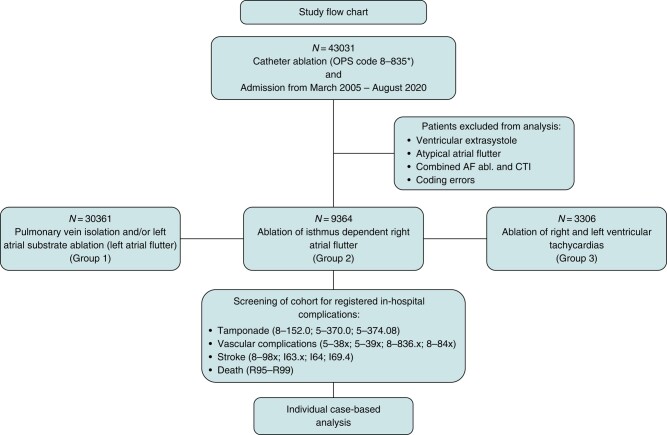
Study flow chart defining in- and exclusion criteria for data extraction and analysis and the overall number and categories of the different kinds of analysed catheter ablations.

Since 2004, diagnoses and procedures must be mandatorily transferred to the German Diagnosis Related Groups system (G-DRG) by all hospital-based health care providers. Diagnoses are coded according to the ICD-10-GM (German Modification), whereas procedures are coded according to the German Operation and Procedure Classification (OPS). Both coding systems allow the precise identification of procedure-related complications during a hospital stay. To ensure uniform results, four centres (University Clinics of Hamburg, Munich, Münster, and Hospital St. George Hamburg) prompted a search in their administrative databases for all catheter ablations from 2005 through 2020 with OPS codes defining the type of ablation and arrhythmias. All ablations for AF, typical AFL, and VT were analysed. To assess the rate of peri-procedural in-hospital complications, we added OPS codes describing (1) the presence of femoral bleeding requiring surgical vascular intervention, (2) a cardiac tamponade, (3) stroke, and (4) in-hospital death. Information on the occurrence of strokes was available from three centres only. Following the administrative query, all patients experiencing at least one of these in-hospital complications were extracted and individually analysed case by case to adjudicate the relation between the ablation procedure and the circumstances of the documented complication (*Figure 1*). Using these ICD and OPS Codes, we classified our patient cohorts in three groups according to the treated arrhythmia (for details, see Supplementary material online, *Appendix Figure S1*):

- Group 1 received a catheter ablation for treatment of AF
- Group 2 was treated for typical AFL by CTI, and
- Group 3 underwent VT ablation.

Group 1 included patients with pulmonary venous isolation (PVI) only and/or additional substrate ablation. Each identified patient with an in-hospital complication was subsequently adjudicated individually for relevance by at least four experienced clinicians (L.E., F.D., M.F.S., G.S.) in the field of cardiac electrophysiology. Cases of in-hospital mortality were further assessed regarding the likelihood of a causal relation between the ablation and the patient's death. The causality of the relation was categorized as ‘likely’, ‘questionable’, and ‘unlikely’.

### Statistical analysis

For descriptive statistics, continuous data are presented as means with standard deviation (SD) or medians with interquartile ranges (IQR). Categorical data are presented as proportions. Normality of data distribution was assessed using the Shapiro–Wilk test. Comparisons between groups were performed using the Pearson’s χ^2^ test for categorical variables, the Student’s *t*-test or the Mann–Whitney *U* test for unpaired continuous variables, and the Wilcoxon rank sum test for paired variables, according to data distribution.

For all tests, two-sided *P*-values of <0.05 were considered statistically significant. The statistical data analysis and visualization was performed using R studio version 3.6.2 (The R Foundation for Statistical Computing, Vienna, Austria). There was no commercial support for conduction of the research or the preparation of this report. Approval was obtained from the ethics committee (2020–872-f-S).

## Results

The participating four ablation centres performed 43 031 catheter ablations from 2005–20, of which 30 361 ablations were treatments of AF, 9364 were CTI ablations for typical AFL, and 3306 ablations for VT. Overall, 594 (1.4%) cases experienced at least one of the defined relevant complications within the same hospital stay of the ablation procedure (*Table 1*). The complication rate was 1.1% (*n* = 325) for AF, 1.0% (*n* = 95) for AFL, and 5.3% (*n* = 175) for patients undergoing VT ablation. Per type of complication, (1) a relevant femoral vascular complication requiring surgical intervention occurred in 0.4% (*n* = 157) of all ablation procedures, (2) a cardiac tamponade occurred in 0.7% (*n* = 300), (3) a stroke in 0.2% (*n* = 65), and (4) death occurred in 0.2% (*n* = 72) of all ablation procedures (*Table 2*). If indicated, uninterrupted anticoagulation was provided in 87.4% of patients undergoing ablation for AF, 58.9% of patients undergoing ablation for AFL, and 41.7% of patients undergoing ablation for VT. Baseline characteristics of all patients, procedural details, and information on adverse events are displayed in *Tables* *1*–*4* and Supplementary material online, Appendix Tables. The number of ablations/per year continuously increased from 2005–20 (*Figure 3*) with three times more AF ablation in 2020 (*n* = 2404) as compared to 2005 (*n* = 817) and 2.5 times increase in VT ablations (2005: *n* = 120; 2020 *n* = 320) (see Supplementary material online, *Appendix Table S7*). Regarding overall complications, a significant increase between 2005 and 2020 was observed (0.76% vs. 1.81%; *P* = 0.004) whereas the complication rate from 2010 or 2015 vs. 2020 was not significantly different (*Table 5*, *Figure 3*, Supplementary material online, *Appendix Tables S7*–*S9*).

**Table 1:** Baseline parameters of patients with a major in-hospital complication after catheter ablation for atrial fibrillation, right atrial isthmus dependent atrial flutter, or ventricular tachycardias

Baseline characteristics					
	Overall	Atrial fibrillation	Atrial flutter	Ventricular tachycardia	*P*-value
Number of complications	594 (1.38)^a^	325 (1.07)	95 (1.01)	175 (5.29)	
Overall number of ablation procedures	43 031	30 361	9364	3306	
Age [years; (IQR)]	69.0 [60.0;73.0]	69.0 [60.8;73.0]	67.0 [57.0;73.0]	69.0 [62.0;74.0]	0.67
Sex (male; %)	362 (61.0)	170 (52.3)	64 (67.4)	128 (73.1)	<0.01
Height [m; (IQR)]	1.8 [1.7;1.8]	1.7 [1.7;1.8]	1.8 [1.7;1.9]	1.8 [1.7;1.8]	<0.01
Weight [kg; (IQR)]	83.0 [72.0;95.0]	82.5 [72.0;93.0]	79.5 [68.0;93.2]	85.0 [74.5;100.0]	0.07
BMI [kg/m^2^; (IQR)]	26.8 [24.3;30.8]	27.1 [24.4;30.8]	25.9 [23.6;28.6]	27.1 [24.7;31.3]	0.05
Hypertension (%)	396 (62.1)	218 (67.1)	66 (69.5)	112 (64.0)	0.78
Diabetes mellitus (%)	102 (17.2)	36 (11.1)	20 (21.1)	46 (26.3)	<0.01
Stroke (%)	58 (9.8)	34 (10.5)	7 (7.4)	17 (9.7)	0.66
Implantable cardioverter defibrillator (ICD; %)	132 (22.2)	16 (4.9)	11 (11.6)	105 (60.0)	<0.01
Nicotine abuse (%)	72 (12.1)	27 (8.3)	16 (16.8)	29 (16.6)	0.02
Non-ischaemic cardiomyopathy (NICM; %)	93 (15.7)	22 (6.8)	17 (17.9)	54 (30.9)	<0.01
Ischaemic cardiomyopathy (ICM; %)	118 (19.9)	14 (4.3)	27 (28.4)	77 (44.0)	<0.01
Peripheral vascular disease (%)	124 (20.9)	51 (15.7)	15 (15.8)	58 (33.1)	<0.01
Chronic obstructive pulmonary disease (%)	41 (6.9)	16 (4.9)	7 (7.4)	18 (10.2)	0.28
Chronic kidney disease (%)	53 (8.9)	20 (6.2)	7 (7.4)	26 (14.8)	<0.01
Obstructive sleep apnoea (%)	40 (6.4)	18 (5.5)	7 (7.4)	15 (8.6)	0.65
CHA_2_DS_2_-VASc score [(IQR)]	3.0 [2.0;4.0]	2.0 [1.0;4.0]	3.0 [2.0;4.0]	3.0 [2.0;4.0]	<0.01
LVEF (% ± SD)	50.7 ± 14.5	55.9 ± 10.0	52.7 ± 15.5	49.7 ± 15.0	<0.01
Peri-procedural medication					
ACE inhibitors (%)	316 (53.2)	160 (49.2)	37 (39.0)	119 (68.0)	<0.01
Beta-blocker (%)	421 (70.9)	247 (76.0)	42 (44.2)	132 (75.4)	<0.01
HMG-CoA reductase inhibitor (%)	189 (31.8)	82 (25.2)	29 (30.5)	78 (44.6)	<0.01
Amiodarone (%)	164 (27.6)	78 (24.0)	18 (19.0)	68 (38.9)	<0.01
Mexiletine (%)	5 (0.8)	0 (0.0)	0 (0.0)	5 (2.9)	<0.01
Oral anticoagulation (%)	413 (69.7)	284 (87.4)	56 (58.9)	73 (41.7)	<0.01
Type of oral anticoagulation					
Phenprocoumon (%)	207 (34.8)	136 (41.9)	28 (29.5)	43 (24.6)	0.20
Apixaban (%)	95 (16.0)	60 (18.5)	13 (13.7)	22 (12.6)	0.65
Dabigatran (%)	16 (2.7)	13 (4.0)	1 (1.1)	2 (1.1)	0.31
Rivaroxaban (%)	80 (13.5)	62 (19.1)	12 (12.6)	6 (3.4)	0.11
Edoxaban (%)	15 (2.5)	13 (4)	2 (2.1)	0 (0.0)	0.27

m, metre; kg, kilograms; LVEF, left ventricular ejection fraction.

^a^One patient who died had an ablation for VT and AFL during the same hospital stay. Thus, the overall number differs from the sum by one patient.

**Table 2:** Major adverse events of patients with an in-hospital complication after catheter ablation for either atrial fibrillation, right atrial isthmus dependent atrial flutter, or ventricular tachycardia

	Overall	Atrial fibrillation	Atrial flutter	Ventricular tachycardia	*P*-value
Number of complications (%)	594 (1.38)^a^	325 (1.07)	95 (1.01)	175 (5.29)	
Total ablation procedures	43 031	30 361	9364	3306	
Timing to complication					
Day of the procedure (%)	373 (62.8)	229 (70.5)	46 (48.4)	98 (56.0)	<0.01
Day after the procedure (%)	91 (15.3)	56 (17.2)	16 (16.8)	19 (10.9)	0.60
>2 days after the procedure (%)	130 (21.9)^a^	40 (12.3)	33 (34.7)	58 (33.1)	<0.01
Type of complications of all catheter ablations					
Vascular intervention/surgery (%)	157 (0.36)	66 (0.22)	49 (0.52)	42 (1.27)	<0.01
Stroke^b^ (%)	65 (0.17)	46 (0.16)	8 (0.13)	11 (0.37)	
Cardiac tamponade (%)	300 (0.70)	202 (0.67)	25 (0.27)	73 (2.20)	<0.01
Death (%)	72 (0.17)^a^	11 (0.04)	13 (0.14)	49 (1.48)	<0.01
Circumstance of complication					
Intra-procedural (%)	239 (40.2)	142 (43.7)	36 (37.9)	61 (35.1)	0.05
Post-procedural (%)	355 (59.8)^a^	183 (56.3)	59 (62.1)	114 (65.1)	0.05

^a^One patient (Patient 2; Supplementary material online, *Appendix Table S6*) who died had an ablation for VT and AFL during the same hospital stay. Thus, the overall number differs from the sum by one patient.

^b^As data and incidences for post-interventional strokes were only available from three out of four participating centres, percentages refer to the total amount of ablation procedures performed by only three centres (*n* = 37 308).

**Table 3:** Individual case analysis of adverse events in 43 031 catheter ablations for either atrial fibrillation, right atrial isthmus dependent atrial flutter, or ventricular tachycardias

	Atrial fibrillation (*n* = 30 361)	Atrial flutter (*n* = 9364)	Ventricular tachycardia (*n* = 3306)	Ventricular tachycardia in structural heart disease	Idiopathic ventricular tachycardia
Femoral vascular complication (%)	63 (0.21)	49 (0.52)	37 (1.12)	28	9
Tamponade (%)	202 (0.67)	25 (0.27)	73 (2.21)	43	30
Stroke^a^ (%)	46 (0.16)	8 (0.13)	11 (0.37)	7	4
Death (%)	9 (0.03)	4 (0.04)	14 (0.42)	12	2

^a^As data and incidences for post-interventional strokes were only available from three out of four participating centres, percentages refer to the total amount of ablation procedures performed by only three centres (*n* = 37 308).

**Table 4:** Baseline characteristics and case description of patients with in-hospital death with likely direct relation to the coded prior catheter ablation of either a ventricular tachycardia, atrial fibrillation, or right isthmus dependent atrial flutter (*n* = 27)

NR	Age (Y)	Gender	Comorbidities	CMP	ICD	LVEF (%)	Antiarrhythmic medication	OAC	LOS	Cause of death
Atrial fibrillation										
1	61	Male	None	NICM	Yes	35	Amiodarone	None	4	Post-interventional cardiac tamponade
2	65	Female	PVD	None	No	69	None	None	20	Cardiogenic shock after esophago-atrial fistula with LA perforation
3	92	Male	PVD	None	No	50	None	Apixaban	15	Post-interventional CPR due to hypoxia-related asphyxia based on a nosocomial pneumonia
4	84	Female	None	NA	No	55	Amiodarone	Rivaroxaban	7	Post-interventional cardiac tamponade
5	69	Female	PVD	None	No	55	None	Phenprocoumon	6	Septic multiorgan failure with possible ablation-associated severe infection
6	79	Female	CKD	None	No	NA	None	Phenprocoumon	4	Death following resuscitation after operation of a post-interventional vascular complication
7	67	Female	None	None	No	55	None	Phenprocoumon	18	CPR due to pulseless electrical activity in the setting of a haemorrhagic shock due to a post-interventional pericardial effusion
8	80	Male	None	NICM	Yes	20	None	Rivaroxaban	23	Post-interventional pulmonary arterial embolism
9	70	Male	CKD	ICM	Yes	40	Amiodarone and mexiletine	None	36	Hypoxic brain injury of unknown cause after ablation
Atrial flutter										
10	38	Male	None	None	No	63	None	None	1	Intra-procedural pericardial tamponade after ablation with cardiogenic shock and death
11	77	Male	None	None	No	60	None	None	6	Post-interventional stroke
12	51	Female	None	ICM	No	54	None	Phenprocoumon	5	Retroperitoneal bleeding after ablation in the presence of intensive oral anticoagulation for mechanical mitral valve
13	86	Male	COPD	None	No	73	None	Apixaban	18	Post-interventional stroke
Ventricular tachycardia										
14	67	Male	PVD, COPD, CKD	ICM	Yes	30	Mexiletine	Phenprocoumon	43	CPR with pulmonary embolism and electro-mechanic uncoupling after VT ablation; 43 days after ablation septic shock
15	49	Male	PVD, CKD	ICM	Yes	12	None	Phenprocoumon	11	Septic shock after acute ischaemia of the popliteal artery with necrosis
16	72	Female	PVD	ICM	No	45	None	None	2	Pulmonary arterial embolism
17	80	Male	None	NICM	Yes	30	Amiodarone	Apixaban	2	Post-interventional cardiac tamponade
18	47	Male	None	NICM	Yes	12	None	Phenprocoumon	12	Post-interventional stroke
19	75	Male	None	ICM	Yes	40	Amiodarone	Phenprocoumon	15	Post-interventional stroke
20	63	Male	NICM	NICM	Yes	30	Amiodarone	Phenprocoumon	10	Aortic puncture at transseptal puncture with fatal haemorrhagic shock
21	64	Male	NA	NA	Yes	NA	Mexiletine	None	6	Intra-procedural pericardial tamponade with surgical management and post-operative sepsis resulting in a combined septic and cardiogenic shock
22	64	Male	None	NICM	No	39	None	None	6	Post-interventional tamponade
23	79	Male	None	NICM	Yes	19	Amiodarone and mexiletine	Phenprocoumon	11	Post-interventional tamponade
24	68	Female	PVD	ICM	Yes	20	Amiodarone	None	2	Post-interventional tamponade
25	89	Male	PVD	ICM	NA	51	Amiodarone	None	NA	Post-interventional death due to severe pneumonia associated sepsis and shock following ablation
26	69	Male	PVD	NICM	Yes	23	Amiodarone	Phenprocoumon	32	Death due to thrombosis with acute leg ischaemia during VT ablation
27	75	Male	PVD	none	No	50	Amiodarone	Phenprocoumon	15	Post-interventional stroke with consequent aspiration pneumonia and sepsis

AFL, right atrial isthmus dependent atrial flutter; CKD, chronic kidney disease; CMP, cardiomyopathy; COPD, chronic obstructive pulmonary disease; CPR, cardiopulmonary resuscitation; ICM, ischaemic cardiomyopathy; LOS, length of stay; LVEF, left ventricular ejection fraction; NA, not available; NICM, non-ischaemic cardiomyopathy; OAC, oral anticoagulation; OHCA, out-of-hospital cardiac arrest; PVD, peripheral vascular disease; VF, ventricular fibrillation; VT, ventricular tachycardia.

**Table 5:** Individual case analysis of adverse events in 43 031 catheter ablations for either atrial fibrillation, right atrial isthmus dependent atrial flutter, or ventricular tachycardias

	AF/AFLA/VT ablations	Overall complications (%)	Femoral vascular complications (%)	Stroke (%)^a^	Iatrogenic tamponade (%)	In-hospital death (%)
2005	1569	12 (0.76)^c^	7 (0.4)	0 (0.0)	5 (0.1)	0 (0.0)
2006	1289	3 (0.23)	1 (0.1)	0 (0.0)	1 (0.1)	1 (0.1)
2007	1437	9 (0.63)	0 (0.0)	1 (0.09)	6 (0.4)	2 (0.1)
2008	1916	18 (0.94)	4 (0.2)	5 (0.3)	8 (0.4)	1 (0.1)
2009	2056	27 (1.31)	7 (0.3)	5 (0.3)	13 (0.6)	2 (0.1)
2010	2001	27 (1.35)^c^	12 (0.6)	2 (0.1)	8 (0.4)	5 (0.2)
2011	3032	39 (1.29)	9 (0.3)	4 (0.1)	20 (0.7)	6 (0.2)
2012	3022	46 (1.52)	16 (0.5)	5 (0.2)	23 (0.8)	2 (0.1)
2013	3236	54 (1.67)	14 (0.4)	5 (0.2)	30 (0.9)	5 (0.2)
2014	3306	51 (1.54)	7 (0.2)	7 (0.2)	33 (1.0)	4 (0.1)
2015	3354	49 (1.46)^c^	7 (0.2)	4 (0.1)	28 (0.8)	10 (0.3)
2016	3404	46 (1.35)	18 (0.5)	3 (0.1)	19 (0.6)	6 (0.2)
2017	3431	42 (1.22)	8 (0.2)	6 (0.2)	22 (0.6)	6 (0.2)
2018	3446	50 (1.45)	12 (0.3)	6 (0.2)	27 (0.8)	5 (0.1)
2019	3215	62 (1.93)	14 (0.4)	6 (0.2)	29 (0.9)	13 (0.4)
2020	3317	60 (1.81)^c^	21 (0.6)	6 (0.2)	28 (0.8)	5 (0.2)
2005–20	43 031	594 (1.38)	157 (0.36)	65 (0.17)	300 (0.70)	72 (0.17)^b^

^a^As data and incidences for post-interventional strokes were only available from three out of four participating centres, percentages refer to the total amount of ablation procedures performed by only three centres each year.

^b^One patient (Patient 2 Supplementary material online, *Appendix Table S6*) who died had an ablation for VT and AFL during the same hospital stay. Thus, the overall number differs from the sum by one patient.

^c^Overall complications were significantly lower in 2005 vs. 2020 (*P* = 0.004) but not significantly different comparing 2010/15 to 2020 (*P* = 0.2 and *P* = 0.25, respectively).

(1) Femoral vascular complication after catheter ablation

Of the 157 patients with a major femoral vascular complication requiring surgical intervention, the rate was 0.2% (*n* = 66) in those undergoing ablation for AF, 0.5% (*n* = 49) in patients undergoing CTI ablation for AFL, and 1.3% (*n* = 42) in those undergoing VT ablation. In 373 patients (62.8%), the femoral vascular complication occurred on the day of the procedure and resulted in a mean length of in-hospital stay of 17.9 ± 13.6 days. In two of these patients, a femoral vascular complication was the adjudicated cause of death during the same hospital stay (*Table 4*). Almost all patients with a femoral vascular complication were on oral anticoagulation (AF *n* = 63, 95.5%; AFL *n* = 35, 71.4%) at the time of hospital admission (see Supplementary material online, *Appendix Tables S1*–*S3*).

Patients receiving a VT ablation presented with a higher comorbidity profile, had a mean CHA_2_DS_2_-Vasc Score of 3 [2.0; 5.0], and had impaired left ventricular function reflected by a left ventricular ejection fraction of 40.9 ± 16.2%. Upon patient-level review, we could document very few cases after VT (*n* = 5) and AF (*n* = 3) ablation, in which a preceding coronary angiogram (not directly related to the ablation procedure) was the most probable cause for the observed major vascular complication, leading to a likely in-hospital ablation-related femoral vascular complication rate of 0.21% vs. 0.22% for AF ablations and 1.1% vs. 1.3% for VT ablations. Arterial puncture for retrograde LV access and/or invasive blood pressure monitoring was performed in 20.4% (*n* = 32) of cases with a femoral complication on the same side where the puncture was performed (AF: *n* = 7, 10.6%, AFL: *n* = 3, 6.1%; VT: *n* = 22; 52.4%).

(2) Cardiac tamponade after catheter ablation

A haemodynamically relevant pericardial effusion necessitating drainage during the same hospital stay occurred in 0.7% (*n* = 202) of AF patients, 0.3% (*n* = 25) of AFL patients, and 2.2% (*n* = 73) of VT patients (*Table 2*). In seven patients (2.3% of those with a tamponade), the ablation-associated tamponade was considered causally related to death [AF: *n* = 2; AFL: *n* = 1; VT: *n* = 4; i.e. 9.7% of all in-hospital deaths (*Table 4*)]. In most patients undergoing AF ablation, the tamponade became haemodynamically relevant intra-procedurally (*n* = 122; 60.4%) (see Supplementary material online, *Appendix Table S1*).

Focusing on VT patients experiencing a tamponade, their median age was 69.0 years [61.0; 73.0] and 64.4% (*n* = 47) were men. The median left ventricular ejection fraction was 46.6 ± 11.4%. The underlying pathology was non-ischaemic cardiomyopathy in 19 patients (26.0%), ischaemic cardiomyopathy in 26 patients (35.6%), and idiopathic VT in the remaining 30 patients (41.1%) (see Supplementary material online, *Appendix Table S3*). In the latter, the mean left ventricular ejection fraction was of 55 ± 13.5%. Ablation in the left ventricle was performed in 40 patients (54.8%) and an epicardial approach was performed in 20 (27.4%). In those with an epicardial approach, only haemodynamically relevant tamponades occurring after pericardial sheath and drain removal were included. Almost all cardiac tamponades after VT ablation required intervention on the day of the procedure (*n* = 71; 97.3%) with 50 (68.5%) patients requiring intra-procedural drainage immediately after catheter removal (see Supplementary material online, *Appendix Table S3*).

(3) In-hospital stroke after catheter ablation

A total of 65 patients (0.2%) suffered a peri-interventional stroke (AF: *n* = 46, 0.2%; AFL: *n* = 8; 0.1%; VT: *n* = 11; 0.4%) (*Table 2*). If an oral anticoagulation was taken at the time of hospital admission, it was paused before the procedure in 60% of these cases. In AF patients with a peri-interventional stroke, oral anticoagulation was paused prior to the ablation in 50% (*n* = 23) of the cases, typically >1 day prior to the procedure (*n* = 22; 47.8%). Most strokes became clinically evident early after the ablation (*n* = 40; 87.0%), either on the day of (*n* = 21; 45.7%) or on the day after the ablation (*n* = 19; 41.3%). Eight patients (0.1%) with AFL (see Supplementary material online, *Appendix Table S2*) had a peri-interventional stroke after CTI ablation. Seven of these patients were on oral anticoagulation at the time of admission (87.5%) with four of these patients (57.1%) interrupting their oral anticoagulation prior to the ablation procedure.

Among VT ablations, 11 patients (0.4%) suffered from a peri-interventional in-hospital stroke (*n* = 7 in patients with structural heart disease; *n* = 4 in patients with idiopathic VT). Of these, eight (72.7%) patients were on oral anticoagulation at admission. Oral anticoagulation was paused prior to the ablation in 75.0% (*n* = 6) of these cases, in the majority > 1 day before the procedure (*n* = 5; 62.5%) (see Supplementary material online, *Appendix Table S3*).

(4) In-hospital mortality after catheter ablation

Overall, 72 out of 43 031 patients (0.17%) patients died during their hospital stay. Of these, 11 (0.04%) died after an AF ablation, 13 (0.14%) died following a CTI ablation for AFL, and 49 (1.48%) died following a VT ablation (*Table 2*). One of the patients who died after the VT procedure had a previous CTI ablation for typical AFL during the same hospital stay (see Supplementary material online, *Appendix Table S6*, Patient 2). This patient was analysed both in the AFL and the VT groups but was counted only once in the overall analysis. Most patients died more than 2 days after their catheter-based treatment of AF (*n* = 5; 45.5%), AFL (*n* = 11; 84.6%), or VT (*n* = 37; 75.5%). Two patients with idiopathic VT died during the hospital stay (*Tables 2* and *3*). Of those who died after VT ablation, 19 patients (38.8%) were initially admitted because of electrical storm and *n* = 12 (24.5%) were still inducible at the end of the ablation.

**Figure 2:**
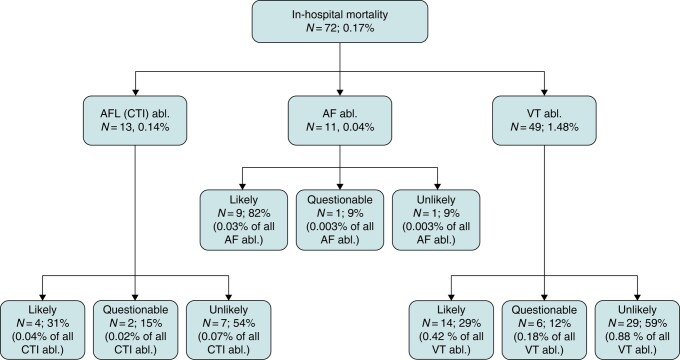
Comparison of in-hospital mortality based on administrative data analysis alone and after adjudication by an individual case inspection. Abl., catheter ablation; AF, atrial fibrillation; AFL, right atrial flutter; CTI, cavo-tricuspid isthmus; VT, ventricular tachycardia.

**Figure 3:**
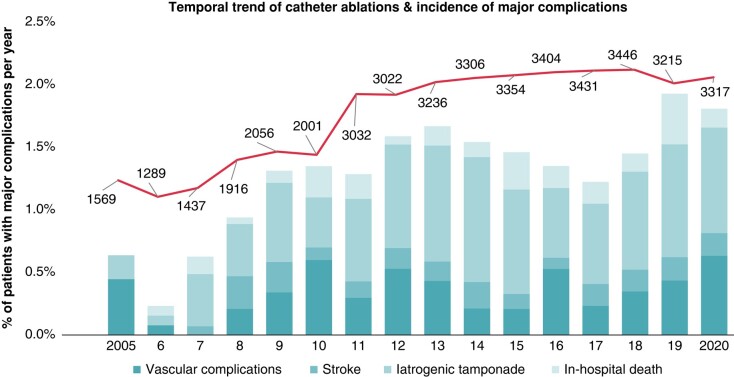
Temporal trend of catheter ablations and incidence of major complications per year from 2005–20.

**Figure 4:**
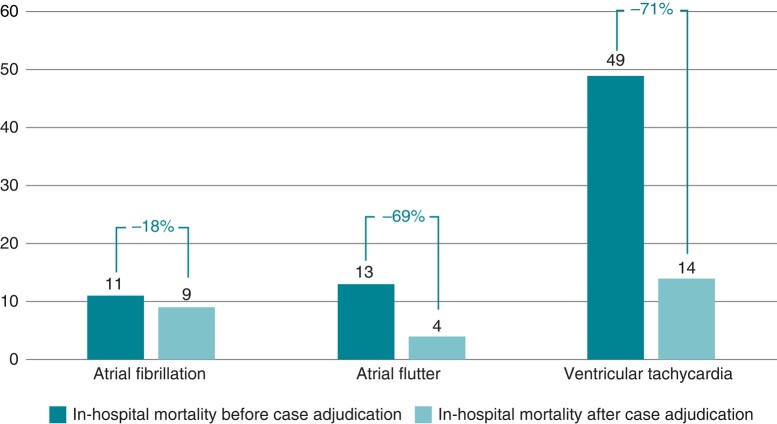
Catheter ablation-related in-hospital mortality based on administrative data analysis alone and after adjudication by an individual case inspection. Percentages indicate the relative change in each group. Atrial flutter: cavo-tricuspid isthmus dependent right atrial flutter.

In contrast to the other reported adverse events, in-hospital mortality was not always related to the ablation procedure itself (*Figures 2* and *3*; Supplementary material online, *Appendix Tables S5* and *S6*). In 27 out of 72 patients (37.5%) who died during the hospital stay, there was a likely association of in-hospital death with the catheter ablation (*Figure 4*, *Table 4*). In contrast, a causal relation between the ablation und death in the same hospital stay was questionable or unlikely in 63.9% (*n* = 46) of deaths. Of all 49 deaths after the ablation of a VT, only 29% (*n* = 14) were adjudicated as directly procedure related. The adjudicated procedure-related mortality rate was hence 0.4% instead of 1.5% by simple counting of administrative data. Similar results were observed for AF with 9 out of 11 deaths (82.0%) and for AFL with 4 out of 13 deaths (30.8%) that were directly related to the catheter ablation (*Tables 2*–*4*, Supplementary material online, *Appendix Tables S5* and *S6*). The resulting adjudicated mortality rates were hence <0.1% for both AF and AFL.

The main causes of ablation-related deaths were thrombo-embolic or haemorrhagic (*Table 4*). Thirty-seven patients (51.4%) were identified with an unlikely association of their in-hospital death with the catheter ablation (see Supplementary material online, *Appendix Table S6*). This included 29 patients with VT ablation, 7 patients with AFL ablation, and 1 patient with ablation for AF. The main reasons for death not related to the ablations included infection (*n* = 12) present before the ablation or occurring late after ablation due to severe comorbidities, malignancies (*n* = 1), cardiac decompensation (*n* = 6), or therapy refractory electrical storm (*n* = 10) (see Supplementary material online, *Table S5*).

## Discussion

Catheter ablation of supraventricular and ventricular tachycardias can be associated with severe complications. Here, we present one of the largest series of an individual case analysis of clinically relevant in-hospital complications including femoral vascular complications requiring surgical therapy, cardiac tamponade requiring drainage, stroke, and death based on 43 031 catheter ablations over 15 years in four high-volume catheter ablation centres. This individual case analysis illustrates a low rate of severe complications with catheter ablations in experienced centres but also reveals how a non-adjudicated analysis of administrative data may inflate estimates. As expected from existing literature, the highest overall incidence of major peri-procedural complications was observed for VT ablations (0.6–11.2%),^15,20–23^ followed by AF ablations (1.2–6.3%),^15,17,18,24,25^ and CTI ablations for AFL (0.1–2.6%).^14,17,26,27^ In line with these data, our analysis of major femoral vascular complications, iatrogenic tamponade, stroke, and in-hospital death revealed similar incidences for AF (1.1%) and AFL (1.0%), but an approximately five-fold higher incidence of major adverse events in patients undergoing VT ablation (5.3%).

In addition, the main findings are the following: (1) in the presence of an increase in complex catheter ablations over time and new technological developments (e.g. single shot devices for AF ablation, contact force technology, improved mapping systems including high density mapping with improved and uninterrupted oral anticoagulation), there was a significant increase in the overall low complication rate/year from 0.8% in 2005% to 1.8% in 2020. (2) All cardiac tamponades (0.7%, 0.3%, and 2.2% for AF, AFL, and VT, respectively) and strokes (0.2%, 0.1%, and 0.4% for AF, AFL, and VT, respectively) found in administrative data were related to the ablation procedures, whereas (3) in-hospital mortality was directly related to the ablation procedures in 82%, 31%, and 29% of all in-hospital deaths after AF, AFL, and VT ablations, respectively (*Figure 4*).

### In-hospital mortality

We found differences in complication rates depending on patient characteristics and the target arrhythmia (*Figure 2*). As expected, VT ablation carries the highest overall complication rate, which is approximately three-fold the rate seen with SVT ablations in a U.S. database analysis.^15^ In-hospital mortality for VT ablations is higher than that of AF or AFL ablations and ranges between 1.3 and 1.8%.^12,13,20–22^ Our results concur with an incidence of 1.5% compared to rates of 0.1% and <0.1% in AFL and AF ablations, respectively. This difference is likely explained by a more advanced stage of the underlying cardiovascular disease, by the comorbidity profile of VT patients, by the complex nature of the ablation procedure itself, and by frequent procedures in the setting of electrical storm. After adjudicating deaths during the in-hospital stay to be likely ablation-related, the mortality rate was <0.1%, <0.1%, and 0.4% for ablations of AF, AFL, and VT, respectively. These numbers illustrate the low ablation-associated in-hospital mortality risk in high-volume centres while still highlighting the relatively higher risk of death in patients undergoing VT ablation. These results are in line with a recent observational study by Lee *et al*.^28^ on 503 patients with impaired left ventricular function (LVEF < 50%) and a procedure-related mortality rate of 0.4%. In our analysis, the main reasons for non-ablation-related deaths were recurrent therapy-refractory VT or electrical storm and decompensated heart failure. There was no single early ablation-associated death in five major randomized multicentre trials on VT ablation during the last six years^29–33^ illustrating procedural safety in other experienced centres. Yet, these finding may indicate selection bias in randomized trials. Adverse events may be more common outside clinical trials, which is illustrated by a 1.8% in-hospital mortality rate in a large U.S. analysis of 9642 VT ablations^15^ and data of the U.S. National Inpatient Sample with an in-hospital mortality of 0.56% in 167 242 AF catheter ablations between 2010 and 2018.^34^ Prolonged ablation procedures, volume overload, and worsening of renal function may contribute to heart failure progression and finally in-hospital mortality. Thus, an indirect effect of the ablation procedure cannot be ruled out. In severely ill patients with advanced heart failure and recurrent VT, it is difficult to differentiate between complications that are indirectly related to the procedure and adverse events related to the natural course of an advanced disease or to inability to prevent VT recurrences by the ablation procedure. If we added questionable ablation-related deaths to the cases with a likely relation, the relevant gap between the individual case analysis and the non-adjudicated administrative data analysis remained. Within the present dataset, we did not observe a clear trend to a change in complications over time. The fact that the number of AF (×2.5) and VT (×3.0) ablations increased more than ablations for AFL (×0.1) may argue for an overall reduced complication rate in the presence of more complex procedures.

The in-hospital mortality of 13 out of 9364 patients (0.14%) undergoing CTI for AFL is unexpectedly higher than the rate of 11 out of 30 361 patients (0.04%) undergoing AF ablation, since ablation for typical AFL is generally considered a rather ‘safe’ ablation. Yet, the 3–4 times higher mortality rate for AFL ablation seen here was also observed in the administrative German-wide analysis by Steinbeck *et al*.^17^ However, individual case analysis in the present dataset eliminated this difference: of 13 deaths following ablation of isthmus dependent right AFL, only four turned out to be likely ablation-related whereas of 11 cases following AF ablation, nine deaths were likely ablation-related. For AFL patients, mostly infections and end-stage heart failure not related to catheter ablation accounted for the deaths.

### In-hospital stroke

Data on the incidences of in-hospital thrombo-embolic complications after catheter ablation of cardiac arrhythmias are scarce and often outdated; particularly as clinical practice, regarding peri-procedural anticoagulation has changed over time. Regarding AF, prior data on peri-interventional incidences of stroke reported up to 7%^14,25,35,36^ and a prospective randomized study by Di Biase *et al*.^37^ documented an incidence of 3.7%. More recent randomized studies including different energy sources and a modern anticoagulation regiment reported even lower stroke rates of 0–0.6%.^38–42^ Due to the right atrial ablation approach in AFL lower incidences of strokes from 0–0.5% were reported after CTI.^15,17,26^ Regarding VT, a single-centre study reported an incidence of 0.7% for thrombo-embolic events after ablation. The recent PAUSE-SCD Trial^31^ demonstrated a slightly higher incidence of 1.7%.

Overall, our individual case analysis revealed low incidences of in-hospital stroke. A possible explanation may be the fact that the reporting centres have large ablation programmes and use comparable standards for peri-interventional anticoagulation management. It is remarkable that in the majority of those who developed a stroke, oral anticoagulation was paused before ablation. Whereas this observation is rather hypothesis generating, it further emphasizes the relevance of continued anticoagulation in patients undergoing catheter ablation. Further prospective trials covering the important topic of peri-procedural anticoagulation would be of great interest.

### In-hospital cardiac tamponade requiring drainage

Pericardial tamponade is considered one of the most life-threatening acute complications in patients undergoing catheter ablation with an overall incidence of 0.7–3.0% reported by prior studies.^12,15,21,22^ The incidence of tamponades in patients with AF ablation in our report is in line with the lower end of the spectrum of prior findings that reported a prevalence between 0.8 and 1.1%.^17,25,42,43^ A nationwide Swedish cohort study from the Swedish Catheter Ablation Registry (2005–19) recently reported that iatrogenic cardiac tamponade was associated with an increased risk of hospitalization for pericarditis but revealed no significant association with mortality.^44^ This is in line with our observation that most patients with an iatrogenic cardiac tamponade experienced no further complications or severe in-hospital sequela. However, in seven patients (2.3% of all tamponades) the tamponade was identified as the cause of death in our analysis.

### In-hospital femoral vascular complications requiring surgical repair

Femoral vascular complications are the most common complications of catheter ablation for cardiac arrhythmias.^14,17^ Incidences vary by the type of the procedure, definition of the complication itself, and by patient characteristics like gender and age.^14,21,26^ Major bleeding due to vascular complications are reported to occur in 0.7–4.7% of ablations for VT^12,13,23^ compared to 0.5–13.0% for AF^14,17,25^ and <4% for typical AFL.^14,15,17,26^ In line with these results, our analysis revealed an incidence of 0.4% for major femoral vascular complications requiring surgical intervention. Only few cases were due to a preceding coronary angiogram (*n* = 8) leading to a ‘true’ incidence of 0.35%. Of note, we observed a low vascular access site complication rate of 0.2% for patients undergoing AF ablation among our high-volume centres. Parikshit *et al*.^45^ compared the rate of access site complications between patients that did or did not receive ultrasound-guided vascular puncture and access. The ultrasound-guided group experienced a markedly lower complication rate of 0.6% vs. 2.5%. As the use of ultrasound was not specified but in general only rarely performed in our study, it may only be speculated if ultrasound-guided vascular access in high-volume centres may further reduce vascular complications. Most patients with a major femoral vascular complication after either the catheter ablation for AF (95.5%), AFL (53.1%), or VT (40.5%) were on continued oral anticoagulation at the time of the procedure. Given the relatively high incidence of femoral vascular complications after ablation of VT (1.1% after individual case inspection), we encourage further research to reduce and prevent undue procedural risks in this growing cohort of patients.

### Limitations

Some considerations are required when interpreting our study. Given the report on patients from four high-volume ablation centres, a certain degree of selection bias is perceivable and may be the reason for the rather low complication rates between 2005 and 2020 irrespective of novel EP technology/ablation strategies. Because of the increase in complex procedures in patients with likely more comorbidities the higher complication rate from 2005 to 2020 seems comprehensible but cannot be explained with certainty. In addition, we identified our patients based on administrative data, which is subject to the risk of coding errors. Yet given that providing both ICD and OPS codes is mandatory and continuously supervised by health insurance providers in Germany, the risk of errors appears limited. Of importance, any cases of over reporting or miscoding detected by the investigators were excluded from further analysis and data on stroke were not available in one centre. The difference of in-hospital death rate between administrative data and individual case analysis of ablation-associated death may call for better patient selection to avoid complex, cost-intensive EP procedures in those who have a fatal outcome. In addition, our analysis was restricted to the in-hospital stay of each patient. Hence, any complication e.g. oesophageal fistula^46–48^ that come to attention only after discharge was not included in this analysis of in-hospital data.

As we did not perform a case analysis of AF patients not experiencing one of the coded complications (*n* = 30 306), we cannot comment on the proportion of AF patients with PVI only vs. PVI plus additional substrate ablation or ablation for atypical flutter in the overall cohort. In addition, we do also not know the number of patients with idiopathic VT vs. structural heart disease associated VT in the overall group of 3306 VT patients. In line with other studies,^12,17,25,45,49^ we defined severe femoral vascular complications by reporting complications requiring surgical intervention that may underestimate other relevant vascular complications (e.g. treated with thrombin injections) and just represent the tip of an iceberg.

## Conclusion

Analysing more than 43 000 catheter ablations, we demonstrate the importance of individual case inspection when analysing administrative data regarding assumed procedure-related complications. Our data of four high-volume centres show low but increasing ablation-related complications between 2005 and 2020. We found an overestimation of mortality rates related to catheter ablation of arrhythmias unless an individual case adjudication is ensured. Following such an individual case inspection, we conclude that (1) AF, AFL, and VT ablation procedures were associated with much lower ablation-related in-hospital mortality rates than reported previously by studies based on administrative data only and that (2) in the presence of an increase of complex procedures over time, the overall mortality rate as well as the rate of other major adverse events remained low.

## Supplementary Material

euad361_Supplementary_Data

## Data Availability

All relevant data are within the manuscript and its supporting information files (supplemental appendix).
