# Supplementary material for: Major in-hospital complications after catheter ablation of cardiac arrhythmias: individual case analysis of 43 031 procedures
Source: Europace. 2023 Dec 15;26(1):euad361. doi: 10.1093/europace/euad361 (PMC10754182; doi:10.1093/europace/euad361)
Supplement: euad361_Supplementary_Data [file euad361_supplementary_data.zip › Supplemental Appendix Tables .docx]

**Supplemental Appendix Table 1:** Baseline parameters, procedural and adverse events of patients receiving a catheter **ablation for atrial fibrillation** with either the in-hospital occurrence of a vascular complication, cardiac tamponade, stroke, or death.

| **Baseline characteristics and procedural data in patients with Atrial Fibrillation** | | | | | | |  |  |  |  |  |  |  |  |  |  |
| --- | --- | --- | --- | --- | --- | --- | --- | --- | --- | --- | --- | --- | --- | --- | --- | --- |
|  | **Overall** | **Vascular Intervention** | **Cardiac Tamponade** | **Stroke** | **In-hospital Death** | **p-value** |  |  |  |  |  |  |  |  |  |  |
| **Baseline Characteristics** | | | | | | |  |  |  |  |  |  |  |  |  |  |
| **N** | **325** | **66** | **202** | **46** | **11** |  |  |  |  |  |  |  |  |  |  |  |
| Age (years, [IQR]) | 69.0 [60.8;73.0] | 70.0 [63.5;74.0] | 69.0 [61.0;74.8] | 68.0 [59.0;72.8] | 70.0 [66.0;79.5] | 0.08 |  |  |  |  |  |  |  |  |  |  |
| Sex (male (%)) | 169 (52.0) | 25 (37.9) | 110 (54.5) | 29 (63.0) | 5 (45.5) | 0.05 |  |  |  |  |  |  |  |  |  |  |
| BMI (kg/m^2^; [IQR]) | 27.1 [24.4;30.8] | 27.7 [24.2;32.2] | 27.8 [25.1;29.4] | 27.9 [24.4;30.5] | 29.4 [26.4;31.1] | 0.54 |  |  |  |  |  |  |  |  |  |  |
| Hypertension (%) | 218 (67.1) | 49 (74.2) | 136 (67.3) | 26 (56.5) | 7 (63.6) | 0.26 |  |  |  |  |  |  |  |  |  |  |
| Diabetes Mellitus (%) | 36 (11.1) | 10 (15.2) | 19 (9.4) | 4 (8.7) | 3 (27.3) | 0.18 |  |  |  |  |  |  |  |  |  |  |
| Stroke (%) | 34 (10.5) | 12 (18.2) | 20 (9.9) | 2 (4.3) | 0 (0.0) | 0.06 |  |  |  |  |  |  |  |  |  |  |
| Implantable Cardioverter Defibrillator (ICD; %) | 16 (4.9) | 7 (10.6) | 6 (3.0) | 1 (2.2) | 2 (18.2) | <0.01 |  |  |  |  |  |  |  |  |  |  |
| Nicotine Abuse (%) | 27 (8.3) | 4 (6.1) | 19 (9.4) | 4 (8.7) | 0 (0.0) | 0.62 |  |  |  |  |  |  |  |  |  |  |
| Non-ischemic Cardiomyopathy (NICM; %) | 22 (6.8) | 3 (4.5) | 12 (5.9) | 4 (8.7) | 3 (27.3) | 0.04 |  |  |  |  |  |  |  |  |  |  |
| Ischemic Cardiomyopathy (ICM; %) | 14 (4.3) | 3 (4.5) | 7 (3.5) | 4 (8.7) | 0 (0.0) | 0.39 |  |  |  |  |  |  |  |  |  |  |
| Peripheral Vascular Disease (%) | 51 (15.7) | 13 (19.7) | 27 (13.4) | 6 (13.0) | 5 (45.5) | 0.03 |  |  |  |  |  |  |  |  |  |  |
| Chronic Obstructive Pulmonary Disease (%) | 16 (4.9) | 3 (4.5) | 11 (5.4) | 1 (2.2) | 1 (9.1) | 0.73 |  |  |  |  |  |  |  |  |  |  |
| Chronic Kidney Disease (%) | 20 (6.2) | 5 (7.6) | 8 (4.0) | 5 (10.9) | 2 (18.2) | 0.01 |  |  |  |  |  |  |  |  |  |  |
| Obstructive Sleep Apnoea (%) | 18 (5.5) | 2 (3.0) | 14 (6.9) | 2 (4.3) | 0 (0.0) | 0.51 |  |  |  |  |  |  |  |  |  |  |
| CHA_2_DS_2_-VASc score [IQR] | 2.0 [1.0;4.0] | 3.0 [2.0;4.0] | 2.0 [1.0;3.8] | 3.0 [1.0;4.0] | 3.0 [2.5;4.0] | 0.02 |  |  |  |  |  |  |  |  |  |  |
| LVEF (% ± SD) | 56.1±9.5 | 57.0±10.6 | 54.8±9.8 | 56.8±9.2 | 45.1±9.7 | <0.01 |  |  |  |  |  |  |  |  |  |  |
| **Periprocedural Medication** | | | | | | |  |  | |  | |  | |  | | **Periprocedural Medication** |
| ACE-Inhibitors (%) | 160 (49.2) | 32 (48.5) | 93 (46.0) | 29 (63.0) | 6 (54.5) | 0.07 |  |  |  |  |  |  |  |  |  |  |
| Betablocker (%) | 247 (76.0) | 43 (65.2) | 154 (76.2) | 39 (84.8) | 11 (100.0) | 0.04 |  |  |  |  |  |  |  |  |  |  |
| HMG-CoA-Reductase-Inhibitor (%) | 82 (25.2) | 22 (33.3) | 43 (21.3) | 14 (30.4) | 3 (27.3) | 0.08 |  |  |  |  |  |  |  |  |  |  |
| Amiodarone (%) | 78 (24.0) | 23 (34.8) | 41 (20.3) | 11 (23.9) | 3 (27.3) | 0.07 |  |  |  |  |  |  |  |  |  |  |
| Mexiletine (%) | 0 (0.0) | 0 (0.0) | 0 (0.0) | 0 (0.0) | 0 (0.0) |  |  |  |  |  |  |  |  |  |  |  |
| Oral Anticoagulation (%) | 284 (87.4) | 63 (95.5) | 174 (86.1) | 41 (89.1) | 6 (54.5) | 0.52 |  |  |  |  |  |  |  |  |  |  |
| **Type of oral anticoagulation** |  | | | | | |  | |  | |  | |  | |  | |
| Phenprocoumon (%) | 136 (41.8) | 34 (51.5) | 78 (38.6) | 21 (45.7) | 3 (27.3) | 0.69 |  |  |  |  |  |  |  |  |  |  |
| Apixaban (%) | 60 (18.5) | 12 (18.2) | 41 (20.3) | 5 (10.9) | 2 (18.2) | 0.47 |  |  |  |  |  |  |  |  |  |  |
| Dabigatran (%) | 13 (4.0) | 2 (3.0) | 8 (4.0) | 3 (6.5) | 0 (0.0) | 0.82 |  |  |  |  |  |  |  |  |  |  |
| Rivaroxaban (%) | 62 (19.1) | 13 (19.7) | 40 (19.8) | 8 (17.4) | 1 (9.1) | 0.82 |  |  |  |  |  |  |  |  |  |  |
| Edoxaban (%) | 13 (4.0) | 2 (3.0) | 7 (3.5) | 4 (8.7) | 0 (0.0) | 0.82 |  |  |  |  |  |  |  |  |  |  |
| **Procedural Data** | | | | | | |  |  | |  | |  | |  | | **Procedural Data** |
| Length of EP (min; ± SD) | 200.4±83.7 | 183.5±102.2 | 203.7±79.5 | 206.9±78.9 | 161.0±78.1 | 0.17 |  |  |  |  |  |  |  |  |  |  |
| Length of Stay (days; ± SD) | 10.4±8.5 | 15.7±11.9 | 9.0±7.2 | 8.9±6.6 | 14.1±9.9 | <0.01 |  |  |  |  |  |  |  |  |  |  |
| **Location and Circumstances of Ablation** |  | | | | | |  | |  | |  | |  | |  | |
| Transseptal Puncture (%) | 325 (100.0) | 66 (100.0) | 202 (100.0) | 46 (100.0) | 11 (100.0) | 0.01 |  |  |  |  |  |  |  |  |  |  |
| Cryo Ablation (%) | 46(14.2) | 9 (13.6) | 30 (14.9) | 4 (8.7) | 3 (27.3) | 0.1 |  |  |  |  |  |  |  |  |  |  |
| RF Ablation (%) | 259 (79.7) | 52 (78.8) | 161(79.7) | 39 (84.8) | 7(63,6) | 0.1 |  |  |  |  |  |  |  |  |  |  |
| Re-Ablation (%) | 105 (32.3) | 15 (22.7) | 70 (34.7) | 16 (34.8) | 4 (36.4) | 0.09 |  |  |  |  |  |  |  |  |  |  |
| Ablation in Left Atrium (%) | 325 (100.0) | 66 (100.0) | 202 (100.0) | 46 (100.0) | 11 (100.0) | 0.01 |  |  |  |  |  |  |  |  |  |  |
| Ablation in Right Atrium (%) | 0 (0.0) | 0 (0.0) | 0 (0.0) | 0 (0.0) | 0 (0.0) |  |  |  |  |  |  |  |  |  |  |  |
| Ablation in Right Ventricle (%) | 0 (0.0) | 0 (0.0) | 0 (0.0) | 0 (0.0) | 0 (0.0) |  |  |  |  |  |  |  |  |  |  |  |
| Ablation in Left Ventricle (%) | 0 (0.0) | 0 (0.0) | 0 (0.0) | 0 (0.0) | 0 (0.0) |  |  |  |  |  |  |  |  |  |  |  |
| Ablation Epicardial (%) | 0 (0.0) | 0 (0.0) | 0 (0.0) | 0 (0.0) | 0 (0.0) |  |  |  |  |  |  |  |  |  |  |  |
| **Adverse events** | | | | | | |  |  |  |  |  |  |  |  |  |  |
| **Timing to Complication** |  | | | | | |  |  |  |  |  |  |  |  |  |  |
| Day of the procedure (%) | 229 (70.5) | 19 (28.8) | 184 (91.1) | 21 (45.7) | 5 (45.5) | <0.01 |  |  |  |  |  |  |  |  |  |  |
| Day after the procedure (%) | 56 (17.2) | 22 (33.3) | 14 (6.9) | 19 (41.3) | 1 (9.1) | <0.01 |  |  |  |  |  |  |  |  |  |  |
| > 2 days after the procedure (%) | 40 (12.3) | 25 (37.9) | 4 (2.0) | 6 (13.0) | 5 (45.5) | <0.01 |  |  |  |  |  |  |  |  |  |  |
| **Circumstance of Complication** |  | | | | | |  |  |  |  |  |  |  |  |  |  |
| Intraprocedural (%) | 142 (43.7) | 11 (16.7) | 122 (60.4) | 7 (15.2) | 2 (18.2) | <0.01 |  |  |  |  |  |  |  |  |  |  |
| Postprocedural (%) | 183 (56.3) | 55 (83.3) | 80 (39.6) | 39 (84.8) | 9 (81.8) | <0.01 |  |  |  |  |  |  |  |  |  |  |

LVEF = Left ventricular Ejection Fraction; SD = Standard Deviation; IQR = Interquartile Range; EP = Electrophysiological Study

**Appendix Table 2:** Baseline parameters, procedural and adverse events of patients receiving a **catheter ablation for right atrial isthmus dependent atrial flutter** with either the in-hospital occurrence of a vascular complication, cardiac tamponade, stroke, or death.

| **Baseline characteristics and procedural data in patients with Atrial Flutter** | | | | | | |
| --- | --- | --- | --- | --- | --- | --- |
|  | **Overall** | **Vascular Intervention** | **Cardiac Tamponade** | **Stroke** | **In-hospital Death** | **p-value** |
| **Baseline Characteristics** | | | | | | |
| **n** | **95** | **49** | **25** | **8** | **13** |  |
| Age (years; [IQR]) | 67.0 [58.5;74.0] | 68.0 [60.0;76.0] | 71.0 [57.0;73.0] | 73.5 [67.5;75.2] | 65.0 [55.0;71.0] | 0.29 |
| Sex (male; (%)) | 65 (68.4) | 31 (63.3) | 16 (64.0) | 6 (75.0) | 12 (92.3) | 0.23 |
| BMI (kg/m^2^; [IQR]) | 26.0 [23.6;29.4] | 26.3 [23.8;29.1] | 26.0 [24.0;28.7] | 35.3 [30.1;37.8] | 23.2 [19.9;26.9] | 0.18 |
| Hypertension (%) | 66 (69.5) | 34 (69.4) | 14 (56.0) | 8 (100.0) | 10 (76.9) | 0.11 |
| Diabetes Mellitus (%) | 20 (21.1) | 8 (16.3) | 4 (16.0) | 4 (50.0) | 4 (30.8) | 0.12 |
| Stroke (%) | 7 (7.4) | 4 (8.2) | 2 (8.0) | 0 (0.0) | 1 (7.7) | 0.87 |
| Implantable Cardioverter Defibrillator (ICD; %) | 11 (11.6) | 6 (12.2) | 4 (16.0) | 0 (0.0) | 1 (7.7) | 0.76 |
| Nicotine Abuse (%) | 16 (16.8) | 5 (10.2) | 4 (16.0) | 2 (25.0) | 5 (38.5) | 0.10 |
| Non-ischemic Cardiomyopathy (NICM; %) | 17 (17.9) | 10 (20.4) | 4 (16.0) | 1 (12.5) | 2 (15.4) | 0.92 |
| Ischemic Cardiomyopathy (ICM; %) | 27 (28.4) | 10 (20.4) | 9 (36.0) | 2 (25.0) | 6 (46.2) | 0.23 |
| Peripheral Vascular Disease (%) | 15 (15.8) | 5 (10.2) | 5 (20.0) | 1 (12.5) | 4 (30.8) | 0.29 |
| Chronic Obstructive Pulmonary Disease (%) | 7 (7.4) | 1 (2.0) | 1 (4.0) | 2 (25.0) | 3 (23.1) | 0.01 |
| Chronic Kidney Disease (%) | 7 (7.4) | 1 (2.0) | 2 (8.0) | 3 (37.5) | 1 (7.7) | 0.01 |
| Obstructive Sleep Apnoea (%) | 7 (7.4) | 2 (4.1) | 2 (8.0) | 3 (37.5) | 0 (0.0) | 0.01 |
| CHA_2_DS_2_-VASc score [IQR] | 3.0 [2.0;4.0] | 3.0 [2.0;3.0] | 3.0 [1.0;4.0] | 5.0 [3.5;5.0] | 4.0 [2.0;6.0] | 0.04 |
| LVEF (%; ± SD) | 52.7±15.5 | 54.9±15.8 | 50.4±16.4 | 52.8±10.2 | 49.7±16.4 | 0.61 |
| **Periprocedural Medication** | | | | | | |
| ACE-Inhibitors (%) | 37 (38.9) | 19 (38.8) | 9 (36.0) | 6 (75.0) | 3 (23.1) | 0.12 |
| Betablocker (%) | 42 (44.2) | 17 (34.7) | 15 (60.0) | 8 (100.0) | 2 (15.4) | <0.01 |
| HMG-CoA-Reductase-Inhibitor (%) | 29 (30.5) | 16 (32.7) | 6 (24.0) | 4 (50.0) | 3 (23.1) | 0.50 |
| Amiodarone (%) | 18 (18.9) | 6 (12.2) | 8 (32.0) | 1 (12.5) | 3 (23.1) | 0.21 |
| Mexiletine (%) | 0 (0.0) | 0 (0.0) | 0 (0.0) | 0 (0.0) | 0 (0.0) |  |
| Oral Anticoagulation (%) | 56 (58.9) | 26 (53.1) | 18 (72.0) | 7 (87.5) | 5 (38.5) | 0.07 |
| **Type of oral anticoagulation** |  | | | | | |
| Phenprocoumon (%) | 28 (29.5) | 16 (32.7) | 8 (32.0) | 2 (25.0) | 2 (15.4) | 0.34 |
| Apixaban (%) | 13 (13.7) | 3 (6.1) | 6 (24.0) | 1 (12.5) | 3 (38.5) | 0.08 |
| Dabigatran (%) | 1 (1.1) | 1 (2.0) | 0 (0.0) | 0 (0.0) | 0 (0.0) |  |
| Rivaroxaban (%) | 12 (12.6) | 4 (8.2) | 4 (16.0) | 4 (50.0) | 0 (0.0) | 0.36 |
| Edoxaban (%) | 2 (2.1) | 2 (4.1) | 0 (0.0) | 0 (0.0) | 0 (0.0) | 0.32 |
| **Procedural Data** | | | | | | |
| Length of EP (min; ± SD) | 136.6±91.6 | 98.2±46.2 | 183.1±82.4 | 170.8±76.8 | 167.0±131.1 | 0.01 |
| Length of Stay (days; ± SD) | 14.4±14.0 | 12.7±7.7 | 8.9±9.0 | 15.5±13.7 | 28.2±26.2 | 0.02 |
| **Location and Circumstances of Ablation** |  | | | | | |
| Transseptal Puncture (%) | 0 (0.0) | 0 (0.0) | 0 (0.0) | 0 (0.0) | 0 (0.0) |  |
| Ablation Method (Cryo; %) | 0 (0.0) | 0 (0.0) | 0 (0.0) | 0 (0.0) | 0 (0.0) |  |
| Re-Ablation (%) | 16 (16.8) | 7 (14.3) | 5 (20.0) | 4 (50.0) | 0 (0.0) | 0.03 |
| Ablation in Left Atrium (%) | 0 (0.0) | 0 (0.0) | 0 (0.0) | 0 (0.0) | 0 (0.0) |  |
| Ablation in Right Atrium (%) | 95 (100.0) | 49 (100.0) | 25 (100.0) | 8 (100.0) | 13 (100.0) |  |
| Ablation in Right Ventricle (%) | 0 (0.0) | 0 (0.0) | 0 (0.0) | 0 (0.0) | 0 (0.0) |  |
| Ablation in Left Ventricle (%) | 0 (0.0) | 0 (0.0) | 0 (0.0) | 0 (0.0) | 0 (0.0) |  |
| Ablation Epicardial (%) | 0 (0.0) | 0 (0.0) | 0 (0.0) | 0 (0.0) | 0 (0.0) |  |
| **Adverse events** | | | | | | |
| **Timing to Complication** |  | | | | | |
| Day of the procedure (%) | 46 (48.4) | 20 (40.8) | 22 (88.0) | 2 (25.0) | 2 (15.4) | <0.01 |
| Day after the procedure (%) | 16 (16.8) | 11 (22.4) | 1 (4.0) | 4 (50.0) | 0 (0.0) | 0.02 |
| > 2 days after the procedure (%) | 33 (34.7) | 18 (36.7) | 2 (8.0) | 2 (25.0) | 11 (84.6) | <0.01 |
| **Circumstance of Complication** |  | | | | | |
| Intraprocedural (%) | 36 (37.9) | 22 (44.9) | 12 (48.0) | 1 (12.5) | 1 (7.7) | 0.02 |
| Postprocedural (%) | 59 (62.1) | 27 (55.1) | 13 (52.0) | 7 (87.5) | 12 (92.3) | 0.12 |

LVEF = Left ventricular Ejection Fraction; SD = Standard Deviation; IQR = Interquartile Range; EP = Electrophysiological Study

**Supplemental Appendix Table 3:** Baseline parameters, procedural and adverse events of patients receiving a **catheter ablation of ventricular tachycardia** with either the in-hospital occurrence of a vascular complication, cardiac tamponade, or death.

| **Baseline characteristics and procedural data in patients with catheter ablation for Ventricular Tachycardias** | | | | | | |
| --- | --- | --- | --- | --- | --- | --- |
|  | **Overall** | **Vascular Intervention** | **Cardiac Tamponade** | **Stroke** | **In-hospital Death** | **p-value** |
| **Baseline Characteristics** | | | | | | |
| **n** | **175** | **42** | **73** | **11** | **49** |  |
| Age (years; [IQR]) | 69.0 [61.0; 74.0] | 69.5 [60.0;78.0] | 69.0 [61.0;73.0] | 61.0 [56.0;73.0] | 69.0 [63.0;74.0] | 0.37 |
| Sex (male; (%)) | 128 (73.1) | 31 (73.8) | 47 (64.4) | 7 (63.6) | 43 (87.8) | 0.02 |
| BMI (kg/m^2^; [IQR]) | 27.1 [24.7;31.3] | 26.8 [24.9;32.9] | 26.3 [23.8;30.3] | 27.7 [25.8;30.7] | 29.5 [26.4;34.2] | 0.17 |
| Hypertension (%) | 112 (64.0) | 27 (64.3) | 49 (67.1) | 5 (45.5) | 31 (63.3) | 0.55 |
| Diabetes Mellitus (%) | 46 (26.3) | 11 (26.2) | 18 (24.7) | 2 (18.2) | 15 (30.6) | 0.59 |
| Stroke (%) | 17 (9.7) | 3 (7.1) | 6 (8.2) | 0 (0.0) | 8 (16.3) | 0.23 |
| Implantable Cardioverter Defibrillator (ICD; %) | 105 (60.0) | 24 (57.1) | 39 (53.4) | 5 (45.5) | 37 (75.5) | 0.01 |
| Nicotine Abuse (%) | 29 (16.6) | 5 (11.9) | 12 (16.4) | 2 (18.2) | 10 (20.4) | 0.73 |
| Non-ischemic Cardiomyopathy (NICM; %) | 51 (29.1) | 9 (21.4) | 19 (26.0) | 2 (45.5) | 21 (42.9) | 0.03 |
| Ischemic Cardiomyopathy (ICM; %) | 76 (43.4) | 22 (52.4) | 26 (35.6) | 5 (54.5) | 23 (46.9) | 0.16 |
| Idiopathic VT (%) | 47 (26.9) | 11 (26.2) | 30 (41.1) | 4 (36.4) | 2 (4.1) | 0.09 |
| Peripheral Vascular Disease (%) | 58 (33.1) | 19 (45.2) | 19 (26.0) | 2 (18.2) | 18 (36.7) | 0.03 |
| Chronic Obstructive pulmonary disease (%) | 18 (10.3) | 1 (2.4) | 8 (11.0) | 3 (27.3) | 6 (12.2) | 0.09 |
| Chronic kidney disease (%) | 26 (14.9) | 5 (11.9) | 10 (13.7) | 2 (18.2) | 9 (18.4) | 0.55 |
| Obstructive Sleep Apnea (%) | 15 (8.6) | 3 (7.1) | 6 (8.2) | 3 (27.3) | 3 (6.1) | 0.15 |
| CHA_2_DS_2_-VASc score [IQR] | 3.0 [2.0;4.0] | 3.0 [2.0;5.0] | 3.0 [2.0;4.0] | 3.0 [1.5;4.5] | 3.0 [3.0;4.0] | 0.54 |
| LVEF (%; ± SD) | 39.7±15.0 | 40.9±16.2 | 46.6±11.4 | 39.4±16.7 | 27.5±10.9 | <0.01 |
| **Periprocedural Medication** | | | | | | |
| ACE-Inhibitors (%) | 119 (68.0) | 23 (54.8) | 53 (72.6) | 7 (63.6) | 36 (73.5) | 0.09 |
| Betablocker (%) | 132 (75.4) | 28 (66.7) | 58 (79.5) | 9 (81.8) | 37 (75.5) | 0.40 |
| HMG-CoA-Reductase-Inhibitor (%) | 78 (44.6) | 17 (40.5) | 31 (42.5) | 6 (54.5) | 24 (49.0) | 0.54 |
| Amiodarone (%) | 68 (38.9) | 15 (35.7) | 24 (32.9) | 2 (18.2) | 27 (55.1) | <0.01 |
| Mexiletine (%) | 5 (2.9) | 1 (2.4) | 2 (2.7) | 0 (0.0) | 2 (4.1) | 0.83 |
| Oral Anticoagulation (%) | 73 (41.7) | 17 (40.5) | 22 (30.1) | 8 (72.7) | 26 (53.1) | 0.02 |
| **Type of oral anticoagulation** |  | | | | | |
| Phenprocoumon (%) | 43 (24.6) | 10 (23.8) | 8 (11.0) | 4 (36.4) | 21 (42.9) | 0.03 |
| Apixaban (%) | 22 (12.6) | 3 (7.1) | 12 (17.8) | 3 (27.3) | 4 (8.2) | 0.02 |
| Dabigatran (%) | 2 (1.1) | 1 (2.4) | 0 (0.0) | 0 (0.0) | 1 (2.0) | 0.41 |
| Rivaroxaban (%) | 6 (3.4) | 3 (7.0) | 2 (2.7) | 1 (9.1) | 0 (0.0) | 0.21 |
| Edoxaban (%) | 0 (0.0) | 0 (0.0) | 0 (0.0) | 0 (0.0) | 0 (0.0) |  |
| **Procedural Data** | | | | | | |
| Length of EP (min; ± SD) | 231.5±104.6 | 196.8±87.8 | 235.1±102.4 | 202.9±109.9 | 256.0±113.2 | 0.13 |
| Length of Stay (days; ± SD) | 28.2±19.8 | 18.8±5.7 | 12.8±9.9 | 18.9±13.3 | 47.7±32.0 | 0.01 |
| **Location and Circumstances of Ablation** |  | | | | | |
| Transseptal Puncture (%) | 113 (64.6) | 32 (76.2) | 42 (57.5) | 7 (63.6) | 32 (65.3) | 0.24 |
| Ablation Method (Cryo; %) | 0 (0.0) | 0 (0.0) | 0 (0.0) | 0 (0.0) | 0 (0.0) |  |
| Re-Ablation (%) | 43 (24.6) | 7 (16.7) | 21 (28.8) | 4 (36.4) | 11 (22.4) | 0.48 |
| Ablation in Left Atrium (%) | 0 (0.0) | 0 (0.0) | 0 (0.0) | 0 (0.0) | 0 (0.0) |  |
| Ablation in Right Atrium (%) | 0 (0.0) | 0 (0.0) | 0 (0.0) | 0 (0.0) | 0 (0.0) |  |
| Ablation in Right Ventricle (%) | 24 (13.7) | 6 (14.3) | 13 (17.8) | 2 (18.2) | 3 (6.1) | 0.51 |
| Ablation in Left Ventricle (%) | 110 (62.9) | 28 (66.7) | 40 (54.8) | 7 (63.6) | 35 (71.4) | 0.24 |
| Ablation Epicardial (%) | 41 (23.4) | 8 (19.0) | 20 (27.4) | 2 (18.2) | 11 (22.4) | 0.19 |
| **Adverse events** | | | | | | |
| **Timing to Complication** |  | | | | | |
| Day of the procedure (%) | 98 (56.0) | 15 (35.7) | 71 (97.3) | 5 (45.5) | 7 (14.3) | <0.01 |
| Day after the procedure (%) | 19 (10.9) | 12 (28.6) | 0 (0.0) | 2 (18.2) | 5 (10.2) | <0.01 |
| > 2 days after the procedure (%) | 58 (33.1) | 15 (35.7) | 2 (2.7) | 4 (36.4) | 37 (75.5) | <0.01 |
| **Circumstance of Complication** |  | | | | | |
| Intraprocedural (%) | 61 (34.9) | 6 (14.3) | 50 (68.5) | 1 (9.1) | 4 (8.2) | <0.01 |
| Postprocedural (%) | 114 (65.1) | 36 (85.7) | 23 (31.5) | 10 (90.1) | 45 (91.8) | <0.01 |

LVEF = Left ventricular Ejection Fraction; SD = Standard Deviation; IQR = Interquartile Range; EP = Electrophysiological Study

***Supplemental Appendix Table 4:*** Procedural Data of patients with an in-hospital complication after catheter ablation of either atrial fibrillation, typical atrial flutter, or ventricular tachycardia (n= 594)

| **Procedural Data** | | | | | |
| --- | --- | --- | --- | --- | --- |
|  | **Overall** | **Atrial fibrillation** | **Atrial flutter** | **Ventricular Tachycardia** | **p-Value** |
|  | **594 (1.48)** * | **325 (1.07)** | **95 (1.01)** | **175 (5.29)** |  |
| Length of procedure (min ± SD) | 201.8±95.7 | 200.4±83.7 | 136.6±91.6 | 231.5±104.6 | <0.01 |
| Length of stay (days ± SD) | 17.9±13.6 | 10.4±8.5 | 14.4±14.0 | 28.2±19.8 | <0.01 |
| **Location and Circumstances of Ablation** | | | | | |
| Transseptal puncture (%) | 393 (66.2) | 325 (100.0) | 0 (0.0) | 113 (64.6) | 0.71 |
| RF ablation (%) [cryo ablation (%)] | 529 (89.1) | 259 (79.7)  [46 (14.2)] | 95 (100.0) | 175 (100.0) | na |
| Re-ablation (%) | 164 (27.6) | 105 (32.3) | 16 (16.8) | 43 (24.6) | 0.25 |
| Ablation in left atrium (%) | 325 (54.7) | 325 (100.0) | 0 (0.0) | 0 (0.0) |  |
| Ablation in Right atrium (%) | 95 (16.0) | 0 (0.0) | 95 (100.0) | 0 (0.0) |  |
| Ablation in Right ventricle (%) | 24 (4.0) | 0 (0.0) | 0 (0.0) | 24 (13.7) |  |
| Ablation in Left ventricle (%) | 110 (18.5) | 0 (0.0) | 0 (0.0) | 110 (62.9) |  |
| Ablation Epicardial (%) | 41 (6.9) | 0 (0.0) | 0 (0.0) | 41 (23.4) |  |

* One patient (Pat. 2 (Supplemental Appendix Table 6) who had an ablation for VT and AFL during the same hospital stay.

**Supplemental Appendix Table 5:** Baseline parameters and case description **of patients with in-hospital demise and questionable relation to the coded prior catheter ablation** (n= 17) of either a ventricular tachycardia, atrial fibrillation, or atrial flutter (n=9)

| NR | Age | Gender | Co-Morbidities | CMP | ICD | LVEF (%) | Antiarrhythmic Medication | OAC | LOS | Cause of Death |
| --- | --- | --- | --- | --- | --- | --- | --- | --- | --- | --- |
| Atrial Flibrillation | | | | | | | | | | |
| 1 | 75 | male | PVD | none | no | 50 | amiodarone | phenprocoumon | 15 | Postinterventional fulminant respiratory insufficiency (ARDS) based on an out of hospital acquired pseudomonas pneumonia |
| Atrial Flutter | | | | | | | | | | |
| 2 | 72 | male | PVD | NICM | na | 30 | amiodarone | apixaban | NA | Postinterventional lung oedema with aggravation of cardiopulmonary status culminating in a cardiogenic shock after ablation |
| 3 | 60 | male | PVD, CKD | NICM | yes | 20 | amiodarone | none | 10 | Primary successful CTI with consequently rapidly progressing cardiomyopathy and death |
| Ventricular tachycardia | | | | | | | | | | |
| 4 | 62 | male | none | ICM | yes | 22 | amiodarone | apixaban | 12 | Therapy refractory electrical storm with therapy refractory VF with prolonged failed CPR at the beginning of EP study |
| 5 | 72 | male | PVD | ICM | yes | 25 | none | none | 8 | Cardiogenic shock due to myocardial failure after multiple electric cardioversions of incessant VT and CPR |
| 6 | 60 | male | none | NICM | na | 20 | amiodarone | none | NA | Recurring VT storms despite amiodarone treatment and death after third VT ablation and 19 external defibrillations due to refractory VF |
| 7 | 70 | male | CKD | ICM | yes | 40 | amiodarone &  mexiletine | none | 26 | Hypoxic brain damage after epicardial ablation and recurrent VF with consequent CPR |
| 8 | 75 | male | none | ICM | yes | 30 | amiodarone | apixaban | 13 | Intraprocedural cardiac arrest during an electrical storm |
| 9 | 65 | male | none | NICM | yes | 30 | none | phenprocoumon | 4 | After ablation cardiac decompensation CPR with elecro-mechanic uncoupling, after ECMO implantation fatal hemorrhagic shock |

CMP= Cardiomyopathy; LVEF = Left ventricular ejection fraction; OAC = Oral Anticoagulation; LOS = Length of Stay; NICM = Non-ischemic Cardiomyopathy; ICM = Ischemic Cardiomyopathy; COPD = Chronic Obstructive Pulmonary Disease; na = not available; VT = ventricular Tachycardia; PVD = Peripheral Vascular Disease; CKD = Chronic Kidney Disease; AF = Atrial Fibrillation; AFL = right atrial isthmus dependent atrial flutter

**Supplemental Appendix Table 6:** Baseline parameters and case description of **patients with in-hospital demise and unlikely relation** to the coded prior catheter ablation of either a ventricular tachycardia, atrial fibrillation, or atrial flutter (n= 37)

| NR | Age | Gender | Co-Morbidities | CMP | ICD | LVEF (%) | Antiarrhythmic Medication | OAC | LOS | Cause of Death |
| --- | --- | --- | --- | --- | --- | --- | --- | --- | --- | --- |
| atrial Fibrillation | | | | | | | | | | |
| 1 | 54 | male | PVD, CKD | NICM | yes | 22 | amiodarone | apixaban | 7 | Cardiogenic shock due to therapy refractory electrical storm |
| atrial Flutter | | | | | | | | | | |
| 2 | 67 | male | PVD | ICM | na | 50 | none | phenprocoumon | NA | Postinterventional ablation cardiac decompensation with lung oedema 30 days after additional VT ablation (i.e. Pat. 10 below) |
| 3 | 68 | male | none | none | no | 64 | none | none | 40 | Sepsis/pneumonia with respiratory failure based on severe co-morbidities |
| 4 | 55 | male | none | ICM | yes | 62 | none | none | 86 | Septic shock due to a necrotizing pancreatitis |
| 5 | 64 | male | none | none | no | na | none | none | 8 | Progressive malignant disease |
| 6 | 54 | male | PVD, COPD, CKD | ICM | no | 50 | amiodarone | apixaban | 20 | Acute respiratory insufficiency, acute kidney injury needing hemodialysis and asystole due to constrictive pericarditis, pneumonia/sepsis and enteritis with clostridia difficile |
| 7 | 71 | male | none | ICM | no | 30 | none | none | na | Death during PCI a week after successful CTI |
| 8 | 65 | male | PVD | ICM | no | 40 | none | none | 52 | Primary septic shock not related to ablation |
| ventricular tachycardia | | | | | | | | | | |
| 9 | 63 | male | PVD | NICM | yes | 25 | amiodarone | none | 9 | Sepsis with multi-organ failure before ablation and bail-out therapy with VT ablation due to an electrical storm and fulminant cardiac decompensation and intraprocedural CPR due to electro-mechanic uncoupling |
| 10 | 67 | male | PVD | ICM | na | 50 | none | phenprocoumon | na | Postinterventional ablation cardiac decompensation with lung oedema 30 days after additional VT ablation (i.e. Pat. 2 above) |
| 11 | 69 | male | none | NICM | yes | 25 | amiodarone | none | 7 | Cardiac arrest due to a progressing NICM |
| 12 | 85 | male | none | NICM | yes | 32 | amiodarone | apixaban | 6 | Electrical storm 7 days after catheter ablation |
| 13 | 48 | female | none | ICM | yes | 14 | amiodarone | dabigatran | 21 | Cardiac Decompensation, pneumonia, HIT with pulmonary embolism |
| 14 | 66 | male | PVD | ICM | yes | NA | none | none | 55 | Cardiac decompensation with severe pulmonary oedema; ablation in electrical storm with decompensated heart failure |
| 15 | 74 | male | none | ICM | yes | 20 | amiodarone | phenprocoumon | 78 | Septic shock due to CRT-device infection |
| 16 | 75 | male | none | NICM | yes | 20 | amiodarone | phenprocoumon | 25 | Decompensated heart failure with respiratory insufficiency |
| 17 | 69 | male | none | NICM | yes | 25 | none | phenprocoumon | 63 | Decompensated heart failure with respiratory insufficiency |
| 18 | 76 | male | none | ICM | yes | 25 | amiodarone | phenprocoumon | 10 | Refractory VT storm despite amiodarone and ablation |
| 19 | 74 | male | none | NICM | yes | na | none | none | 10 | Sepsis with therapy refractory pneumonia |
| 20 | 62 | male | none | NICM | yes | 20 | none | none | 77 | Cardiogenic shock due to a therapy refractory electrical storm with recurring ICD Shocks and CPR |
| 21 | 60 | male | CKD | NICM | yes | 15 | amiodarone | phenprocoumon | 14 | Cessation of LVAD-therapy due to a progressive heart failure |
| 22 | 37 | female | CKD | ICM | no | 20 | none | none | 30 | Cardiogenic shock due to therapy refractory electrical storm |
| 23 | 73 | male | none | ICM | no | 40 | amiodarone | none | 40 | Sepsis due to a S. aureus Infection 40 days after the catheter ablation with multiple co-morbidities |
| 24 | 75 | male | PVD, COPD | NICM | yes | na | amiodarone | phenprocoumon | 96 | Cardiogenic shock due to therapy refractory electrical storm |
| 25 | 47 | male | PCD, CKD | NICM | yes | 10 | amiodarone | none | 285 | Sepsis with acute kidney transplant failure |
| 26 | 57 | male | none | ICM | yes | 20 | none | phenprocoumon | 2 | Therapy refractory electrical storm after out of hospital cardiac arrest |
| 27 | 74 | male | PVD, CKD | ICM | yes | 27 | amiodarone | none | 19 | Cardiogenic shock due to therapy refractory electrical storm |
| 28 | 69 | male | PVD, CKD | none | Yes | 50 | amiodarone | phenprocoumon | 50 | Successful epicardial VT ablation with consequently aggravating clinical status and cardiorenal syndrome needing hemodialysis, hereafter pneumothorax after Sheldon-catheter placement as well as pericarditis with pericardial tamponade needing open heart surgery, hereafter S. aureus sepsis and refractory septic and cardiogenic shock due to infection and pericardial tamponade |
| 29 | 70 | male | PVD | NICM | no | 35 | none | none | 10 | End-Stage Heart Failure with recurring VT after in-hospital STEMI and ne diagnosis of a 3-Vessel coronary artery disease |
| 30 | 64 | male | none | NICM | yes | 20 | none | phenprocoumon | 102 | Progressive right ventricular failure |
| 31 | 69 | male | CKD | NICM | no | 50 | none | none | 8 | Cardiac decompensation a week after catheter ablation |
| 32 | 58 | male | PVD | ICM | yes | 10 | amiodarone | apixaban | na | Recurring CPR due to incessant VT despite VT ablation, antiarrhythmic therapy, and emergency CABP operation with subsequent cardiogenic shock despite ECMO therapy |
| 33 | 65 | male | PVD | ICM | yes | 20 | amiodarone | phenprocoumon | na | ECMO implantation due to VT related cardiogenic shock, after VT ablation superinfection of ECMO cannula culminating in septic shock and death |
| 34 | 81 | male | CKD | NICM | no | 30 | none | phenprocoumon | 74 | Septic/cardiogenic shock > 50 days after ablation |
| 35 | 63 | female | PVD | ICM | yes | 30 | amiodarone | none | 21 | Cardiogenic shock already with LVAD present before ablation, after successful ablation progressive respiratory failure with consequent ECMO implantation, lastly needing explantation due to bleeding of bronchial tree resulting in best supportive care and multi-organ failure |
| 36 | 74 | male | none | ICM | yes | na | none | none | 4 | Pre-Interventional aepsis progressing unrelated to underlying VT-Ablation |
| 37 | 73 | male | PVD | ICM | yes | 35 | amiodarone | phenprocoumon | 46 | Cardiogenic shock due to therapy refractory electrical storm |

CMP= Cardiomyopathy; LVEF = Left ventricular ejection fraction; OAC = Oral Anticoagulation; LOS = Length of Stay; NICM = Non-ischemic Cardiomyopathy; ICM = Ischemic Cardiomyopathy; COPD = Chronic obstructive pulmonary disease; NA = not available; VT = ventricular Tachycardia, PVD = Peripheral Vascular Disease; CKD = chronic kidney disease; AF = Atrial Fibrillation; AFL = Atrial Flutter

***Supplemental Appendix Table 7:*** Individual case analysis of adverse events in 30361 catheter ablations for atrial fibrillation.

|  | **AF**  **Ablations** | **Vascular Femoral Complications (%)** | **Stroke ^#^ (%)** | **Iatrogenic Tamponade (%)** | **In-Hospital Death (%)** |
| --- | --- | --- | --- | --- | --- |
| 2005 | 817 | 1 (0.1) | 0 (0.0) | 2 (0.2) | 0 (0.0) |
| 2006 | 752 | 1 (0.1) | 0 (0.0) | 1 (0.1) | 0 (0.0) |
| 2007 | 836 | 0 (0.0) | 0 (0.0) | 4 (0.5) | 0 (0.0) |
| 2008 | 1299 | 1 (0.1) | 5 (0.4) | 6 (0.5) | 0 (0.0) |
| 2009 | 1336 | 5 (0.4) | 4 (0.3) | 12 (0.9) | 0 (0.0) |
| 2010 | 1341 | 7 (0.5) | 2 (0.2) | 8 (0.6) | 1 (0.1) |
| 2011 | 2170 | 5 (0.2) | 2 (0.1) | 13 (0.6) | 2 (0.1) |
| 2012 | 2178 | 9 (0.4) | 4 (0.2) | 18 (0.8) | 2 (0.1) |
| 2013 | 2395 | 7 (0.3) | 4 (0.2) | 21 (0.9) | 1 (0.04) |
| 2014 | 2418 | 1 (0.04) | 5 (0.2) | 22 (0.9) | 1 (0.04) |
| 2015 | 2458 | 1 (0.4) | 3 (0.1) | 17 (0.7) | 1 (0.04) |
| 2016 | 2565 | 10 (0.4) | 2 (0.1) | 13 (0.5) | 0 (0.0) |
| 2017 | 2542 | 3 (0.1) | 2 (0.1) | 14 (0.6) | 1 (0.04) |
| 2018 | 2551 | 7 (0.3) | 5 (0.2) | 16 (0.6) | 1 (0.04) |
| 2019 | 2299 | 5 (0.2) | 5 (0.2) | 19 (0.8) | 1 (0.04) |
| 2020 | 2404 | 3 (0.1) | 3 (0.1) | 16 (0.7) | 0 (0.0) |
|  |  |  |  |  |  |
| 2005-2020 | 30361 | 66 (0.22) | 46 (0.16) | 202 (0.67) | 11(0.04) |

^#^As Data and incidences for postinterventional strokes were only available from three out of four participating centers, percentages refer to the total amount of AF ablation procedures performed by only three centers (n=28048).

***Supplemental Appendix Table 8:*** Individual case analysis of adverse events in 9364 catheter ablations for right atrial isthmus dependent atrial flutter.

|  | **AFLA**  **Ablations** | **Vascular Femoral Complications (%)** | **Stroke ^#^ (%)** | **Iatrogenic Tamponade (%)** | **In-Hospital Death (%)** |
| --- | --- | --- | --- | --- | --- |
| 2005 | 554 | 6 (1.1) | 0 (0.0) | 2 (0.4) | 0 (0.0) |
| 2006 | 452 | 0 (0.0) | 0 (0.0) | 0 (0.0) | 1 (0.2) |
| 2007 | 482 | 0 (0.0) | 1 (0.5) | 1 (0.2) | 0 (0.0) |
| 2008 | 504 | 1 (0.2) | 0 (0.0) | 1 (0.2) | 0 (0.0) |
| 2009 | 546 | 2 (0.4) | 0 (0.0) | 0 (0.0) | 1 (0.2) |
| 2010 | 493 | 2 (0.4) | 0 (0.0) | 0 (0.0) | 2 (0.4) |
| 2011 | 637 | 2 (0.3) | 1 (0.2) | 1 (0.2) | 0 (0.0) |
| 2012 | 602 | 2 (0.3) | 0 (0.0) | 0 (0.0) | 0 (0.0) |
| 2013 | 632 | 7 (1.1) | 0 (0.0) | 1 (0.2) | 1 (0.2) |
| 2014 | 655 | 3 (0.5) | 0 (0.0) | 3 (0.5) | 0 (0.0) |
| 2015 | 632 | 1 (0.2) | 0 (0.0) | 4 (0.6) | 1 (0.2) |
| 2016 | 613 | 3 (0.5) | 1 (0.2) | 1 (0.2) | 2 (0.3) |
| 2017 | 676 | 2 (0.3) | 2 (0.4) | 1 (0.1) | 2 (0.3) |
| 2018 | 649 | 1 (0.2) | 1 (0.2) | 1 (0.2) | 0 (0.0) |
| 2019 | 625 | 2 (0.3) | 1 (0.2) | 4 (0.6) | 2 (0.3) |
| 2020 | 612 | 15 (2.5) | 1 (0.2) | 5 (0.8) | 1 (0.2) |
|  |  |  |  |  |  |
| 2005-2020 | 9364 | 49 (0.56) | 8 (0.13) | 25 (0.27) | 13 (0.13) |

^#^As Data and incidences for postinterventional strokes were only available from three out of four participating centers, percentages refer to the total amount of AFL ablation procedures performed by only three centers (n=6253).

***Supplemental Appendix Table 9:*** Individual case analysis of adverse events in 3306 catheter ablations for VT.

|  | **VT**  **Ablations** | **Vascular Femoral Complications (%)** | **Stroke ^#^ (%)** | **Iatrogenic Tamponade (%)** | **In-Hospital Death (%)** |
| --- | --- | --- | --- | --- | --- |
| 2005 | 120 | 0 (0.0) | 0 (0.0) | 1 (0.5) | 0 (0.0) |
| 2006 | 163 | 0 (0.0) | 0 (0.0) | 0 (0.0) | 0 (0.0) |
| 2007 | 119 | 0 (0.0) | 0 (0.0) | 1 (0.8) | 2 (1.7) |
| 2008 | 113 | 2 (1.8) | 0 (0.0) | 1 (0.9) | 1 (0.9) |
| 2009 | 174 | 0 (0.0) | 1 (0.6) | 1 (0.6) | 1 (0.6) |
| 2010 | 167 | 3 (1.8) | 0 (0.0) | 0 (0.0) | 2 (1.2) |
| 2011 | 225 | 2 (0.9) | 1 (0.4) | 6 (2.7) | 4 (1.8) |
| 2012 | 242 | 5 (2.1) | 1 (0.4) | 5 (2.1) | 0 (0.0) |
| 2013 | 209 | 0 (0.0) | 1 (0.5) | 8 (3.8) | 3 (1.4) |
| 2014 | 233 | 3 (1.3) | 2 (0.9) | 8 (3.4) | 3 (1.3) |
| 2015 | 264 | 5 (1.9) | 1 (0.5) | 7 (2.7) | 8 (3.0) |
| 2016 | 226 | 5 (2.2) | 0 (0.0) | 5 (2.2) | 4 (1.8) |
| 2017 | 213 | 3 (1.4) | 2 (1.2) | 7 (3.3) | 3 (1.4) |
| 2018 | 246 | 4 (1.6 | 0 (0.0) | 10 (4.1) | 4 (1.6) |
| 2019 | 291 | 7 (2.4) | 0 (0.0) | 6 (2.1) | 10 (3.4) |
| 2020 | 301 | 3 (1.0) | 2 (0.7) | 7 (2.3) | 4 (1.3) |
|  |  |  |  |  |  |
| 2005-2020 | 3306 | 42 (1.27) | 11 (0.37) | 73 (2.2) | 49 (1.48) |

^#^As Data and incidences for postinterventional strokes were only available from three out of four participating centers, percentages refer to the total amount of VT ablation procedures performed by only three centers (n=3007).
